# Supplementary material for: The assembly platform FimD is required to obtain the most stable quaternary structure of type 1 pili
Source: Nat Commun. 2024 Apr 8;15:3032. doi: 10.1038/s41467-024-47212-9 (PMC11001860; doi:10.1038/s41467-024-47212-9)
Supplement: Supplementary file 1 — Supplementary Information [file 41467_2024_47212_MOESM1_ESM.pdf]

**Supplementary Information**

# The assembly platform FimD is required to obtain the most stable quaternary structure of type 1 pili

Dawid S. Zyla[1,$], Thomas Wiegand[2,3,4], Paul Bachmann[1], Rafal Zdanowicz[1], Christoph Giese[1], Beat H. Meier[2], Gabriel Waksman[5], Manuela K. Hospenthal[1,5,*] and Rudi Glockshuber[1]

[1] Institute of Molecular Biology and Biophysics, ETH Zürich, Otto-Stern-Weg 5, 8093 Zürich, Switzerland.

[$] Current address: La Jolla Institute for Immunology, 9420 Athena Cir, La Jolla, CA 92037, United States of America

[2] Laboratory of Physical Chemistry, ETH Zürich, Vladimir-Prelog-Weg 1-5/10, 8093 Zürich, Switzerland.

[3] Institute of Technical and Macromolecular Chemistry, RWTH Aachen University, Worringerweg 2, 52074 Aachen, Germany

[4] Max Planck Institute for Chemical Energy Conversion, Stiftstr. 34-36, 45470 Mülheim/Ruhr, Germany

[5] Institute of Structural and Molecular Biology, University College London and Birkbeck, London WC1E 7HX, United Kingdom.

* Correspondence: manuela.hospenthal@mol.biol.ethz.ch

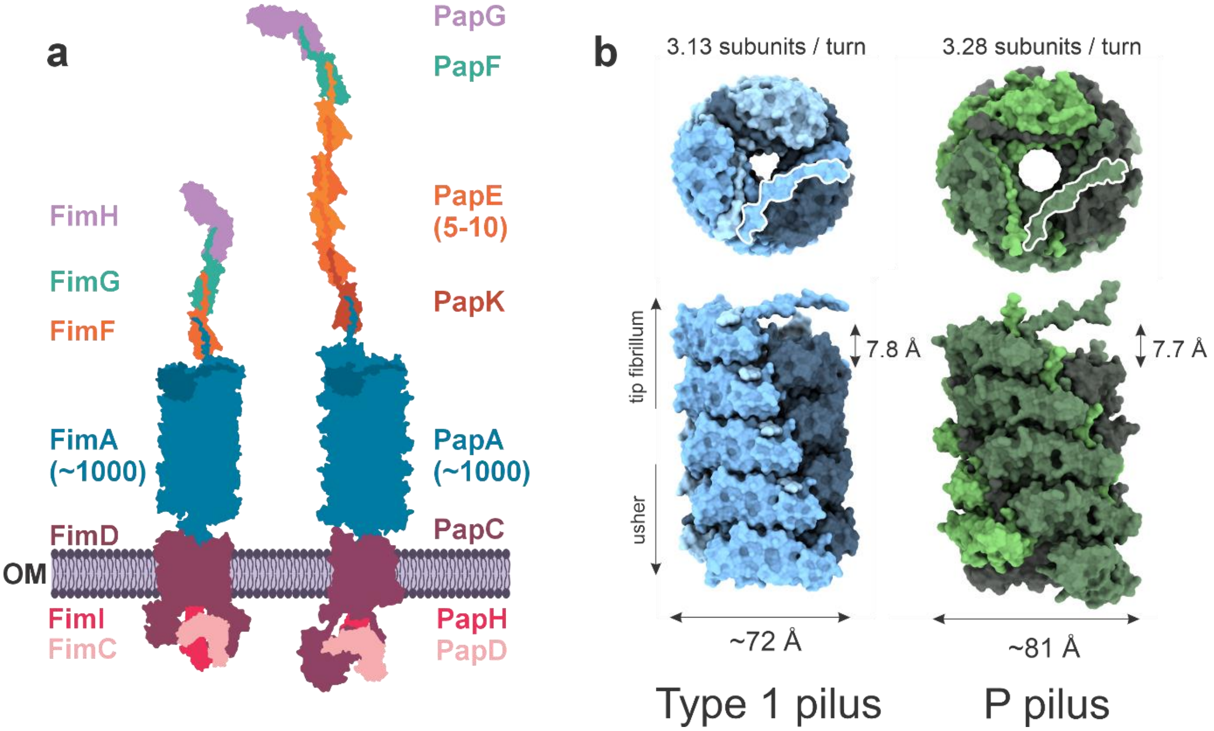


**Fig. S1: Architecture of type 1 and P pili.**

**a** Schematic representation of type 1 pili (left) and P pili (right) assembled at the outer membrane (OM) by the FimD and PapC ushers, respectively. All subunits constituting type 1 and P pili are indicated and coloured differently. **b** Surface representation of type 1 (blue) and P pilus rods (green). The helical rise per subunit, number of subunits per turn, diameter and rod polarity are indicated.

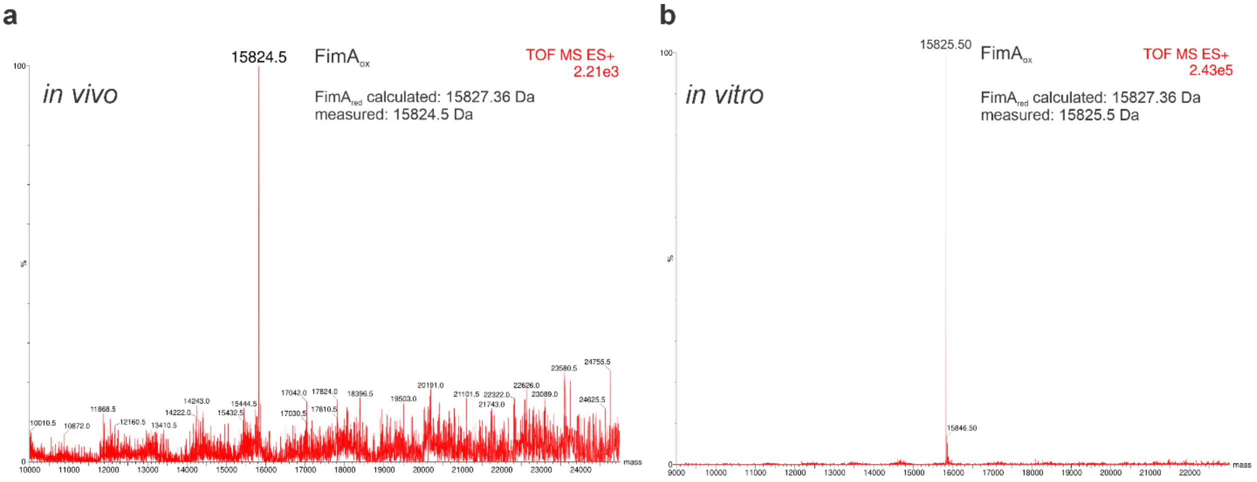


**Fig. S2: Mass spectra of FimA subunits from *in vivo* and *in vitro*-assembled pilus rods.**

**a, b** Intact mass measurements of **(a)** *in vivo* and **(b)** *in vitro*-assembled pilus rods. The experimentally determined masses were 2 Da less than that calculated for the reduced (dithiol) form of FimA, indicating that the single, structural disulphide bond in FimA had been quantitatively formed in both samples**.**

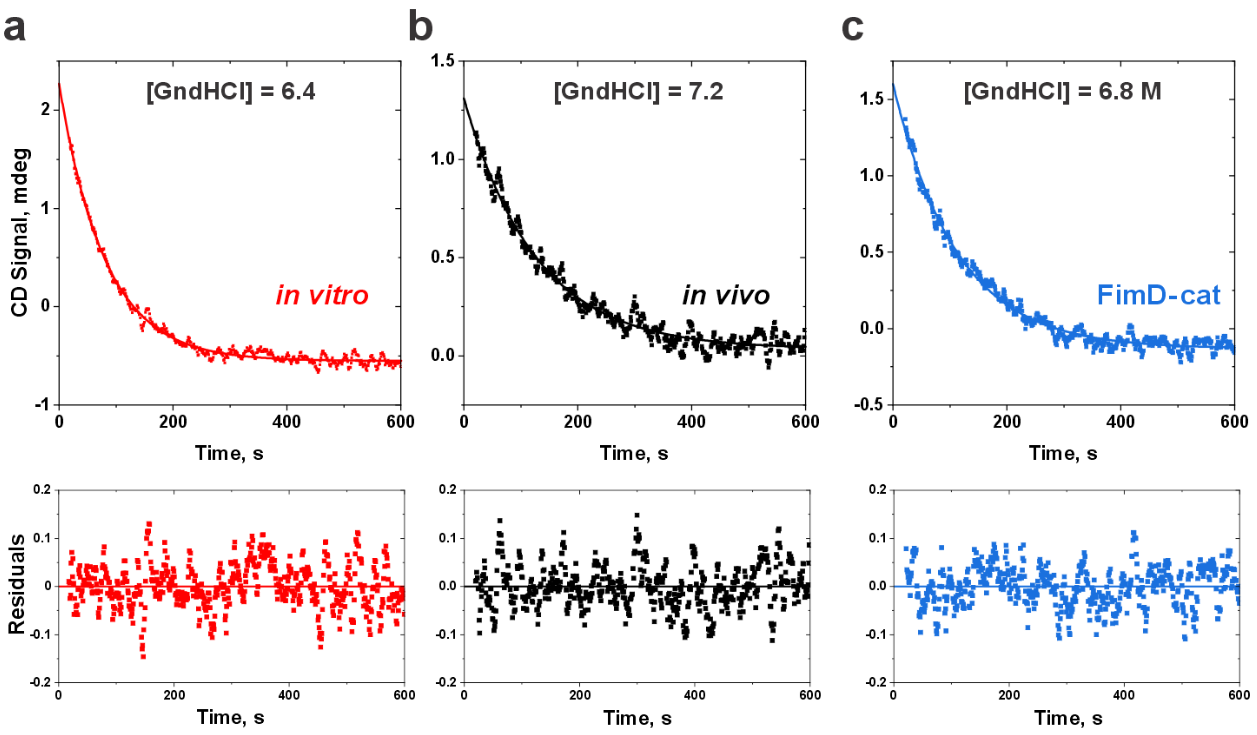


**Fig. S3: Denaturant-induced dissociation/unfolding kinetics of different pilus rod reparations.**

**a-c** Comparison of the unfolding traces and their residuals for **(a)** *in vitro*-assembled, **(b)** *in vivo*-assembled and **(c)** FimD-cat pili recorded via the decrease in the CD signal at 230 nm at different GdmCl concentrations to obtain similar unfolding half-lives. All pilus samples show uniform single exponential kinetics during unfolding (solid lines: fits according to a single mono-exponential decay).

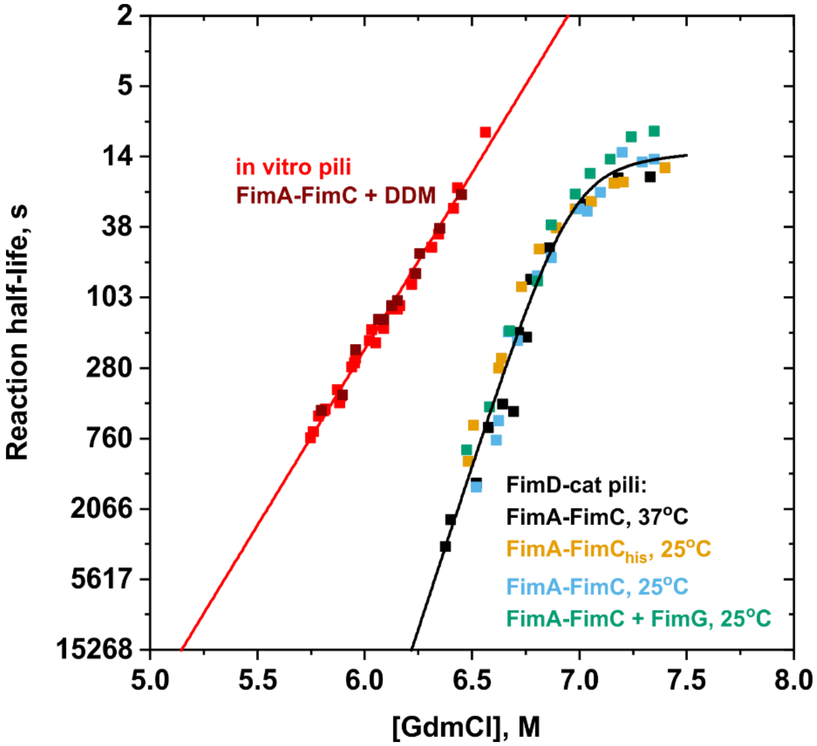


**Fig. S4: Comparison of in vitro-assembled and FimD-cat pili stability under different conditions.**

Dependence of unfolding half-life on GdmCl concentration for *in vitro* pili assembled from FimA-FimC complexes (red, **Fig. 2c**) compared to the same sample in the presence of 0.05% DDM (dark red). Analogous plots are shown for the following FimD-cat pili assembled from FimA-FimC or FimA-$FimC_{his}$: FimA-FimC complexes assembled at 37°C (black), FimA-FimC complexes assembled at 25°C (blue), and FimA-$FimC_{his}$ complexes assembled at 25°C (yellow). All FimD-cat pili were assembled by preactivating the FimDCH complex with FimC-FimF, except one reaction where the complex was preactivated with FimC-FimG instead (green; indicated).

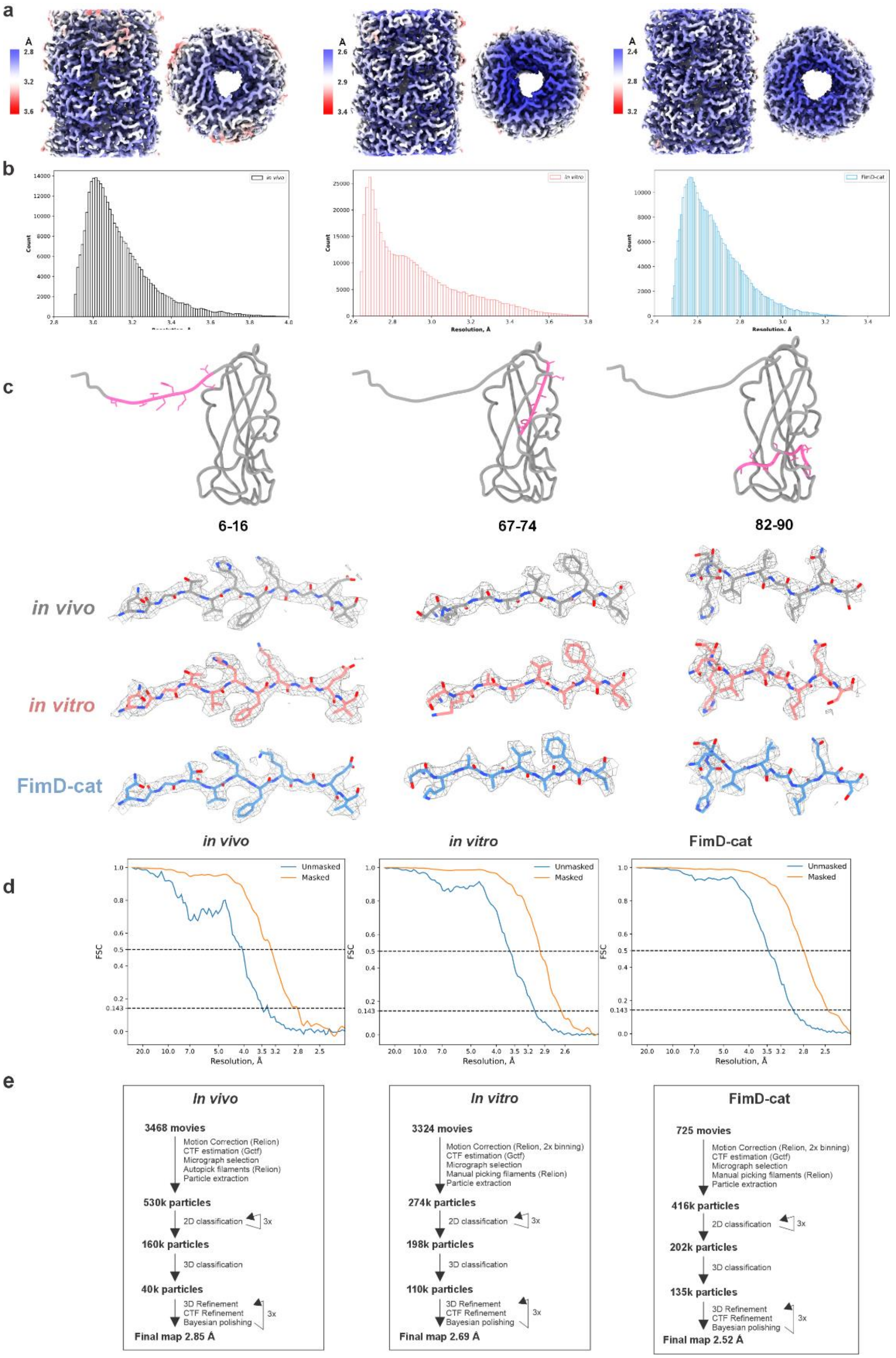


## Fig. S5: Quality assessment of cryo-EM structures.

**a** Local resolution estimation for the *in vivo*-assembled (left), *in vitro*-assembled (middle) and FimD-cat (right) pilus rod structures shown in side and top views. Highest resolution, blue; lowest resolution, red. **b**

Local resolution histograms showing resolution distribution in the masked maps. **c** Comparison of regions with the best local resolution, corresponding to the donor strand (left) and an internal strand (centre), and worst local resolution, corresponding to an outward-facing loop (right). The cryo-EM maps are shown as a grey mesh and the models are shown in stick representation. **d** Fourier shell correlation (FSC) plots calculated for the unmasked and masked maps obtained for the *in vivo*-assembled (left), *in vitro*-assembled (centre) and FimD-cat (right) pilus rod reconstructions. The resolutions of the masked maps at FSC=0.143 were 2.9 Å, 2.7 Å and 2.5 Å for the *in vivo*-assembled, *in vitro*-assembled and FimD-cat structures, respectively. **e** Cryo-EM data processing workflow for all pilus rod structures determined.

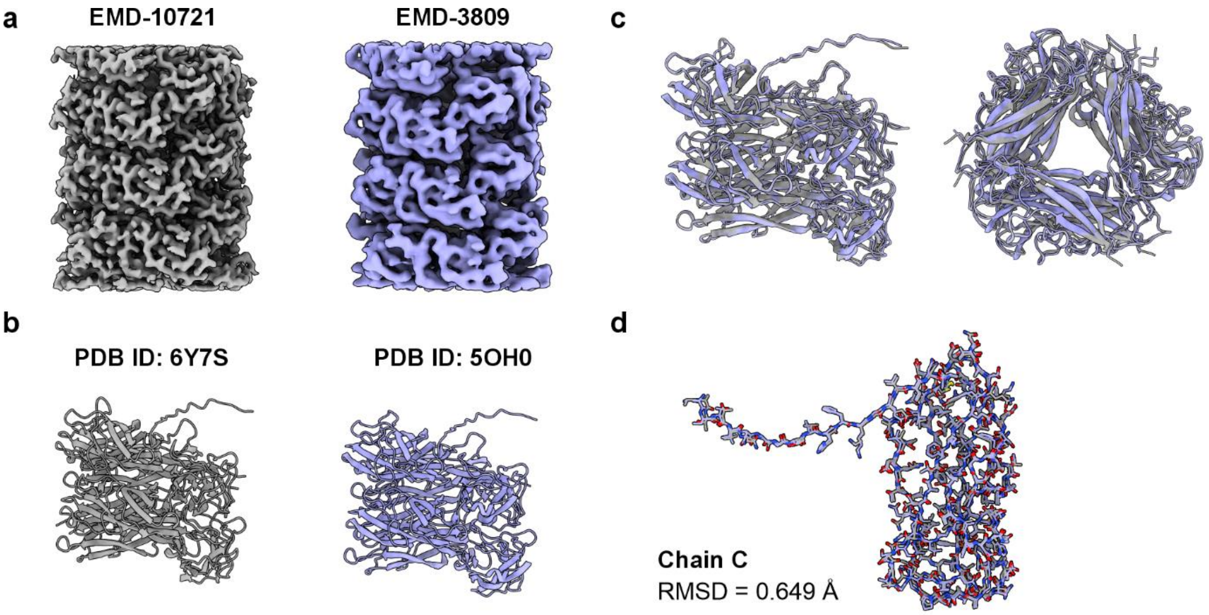


**Fig. S6: Comparative analysis of *in vivo*-assembled type 1 pilus rod structures.**

**a** Cryo-EM maps of the type 1 pilus rod structures determined in the present (grey; EMD-10721) and our previous study (purple, EMD-3809 [1]), resolved to 2.85 Å and 4.20 Å respectively. **b** Corresponding models of the present (grey; PDB ID: 6Y7S) and previously determined (purple; PDB ID: 5OH0 [1]) structures. **c** Superposition of the two models in side (left) and top (right) view. **d** Detailed comparison using stick representation of the central subunit (chain C) from both structures, with an overall Cα RMSD of 0.649 Å.

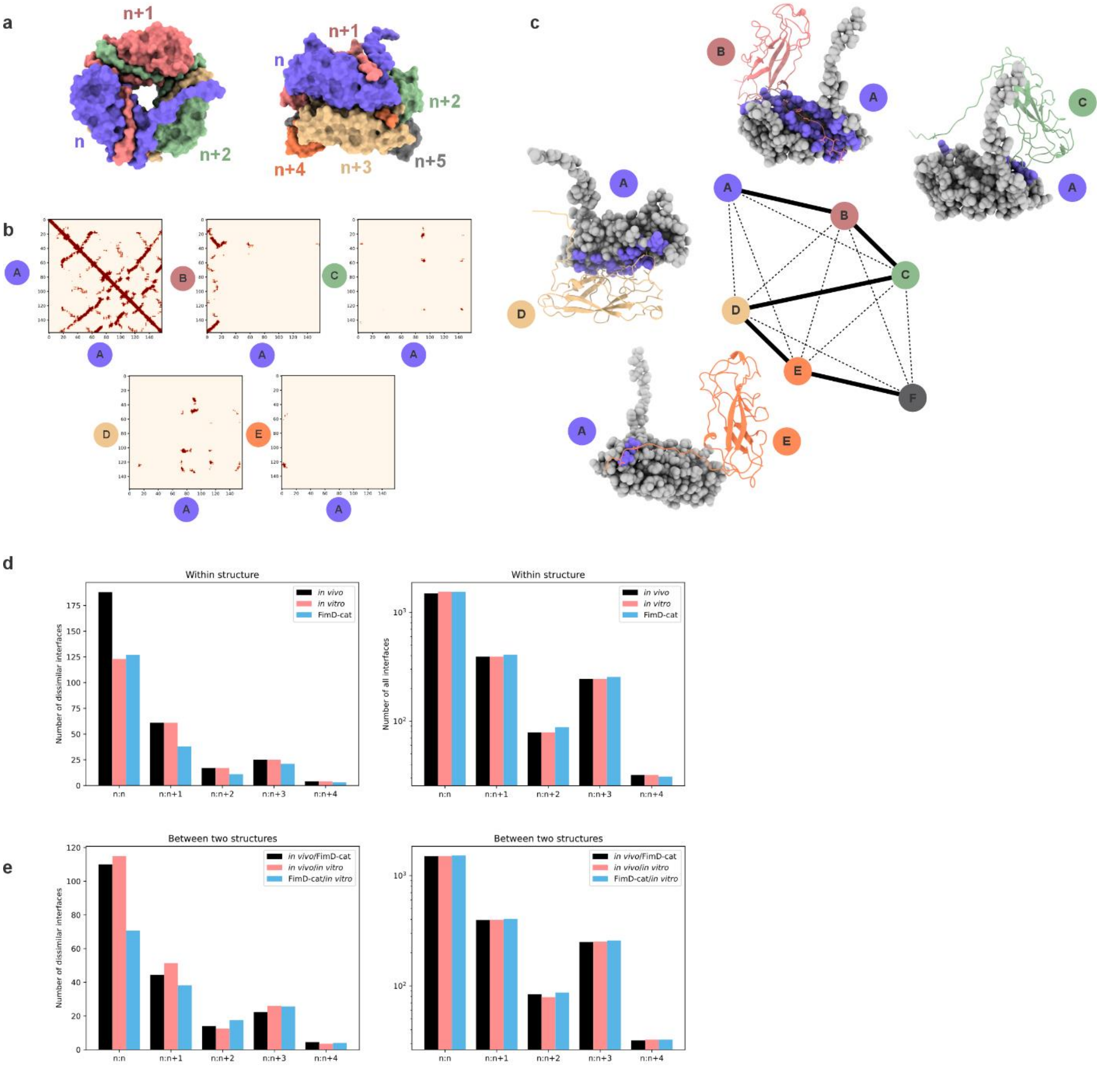


**Fig. S7: Comparison of subunit interfaces within and between cryo-EM structures.**

**a** Pilus rod surface model with labelled FimA subunits. **b** Binary contact maps showing all interfaces present within a pilus structure. The contact map was calculated for residue interactions within 8 Å, and residues within an 8 Å distance are represented as a dot. **c** Graphical representation of interactions present in the pilus rod with highlighted residues interacting between chain A (blue) and 4 other subunits (B: red, C: green, D: pale orange, E: orange). **d** Comparison of interactions in the interfaces, including interactions within a single subunit. Interactions within a single structure differ because pilus rod models were refined without imposing symmetry resulting in slightly different residue conformations in each subunit. Left,

distinct interactions; right, all interactions (log scale on y-axis). **e** Pair-wise comparison of the same interfaces between two structures shows a similar trend as in **(d)**.

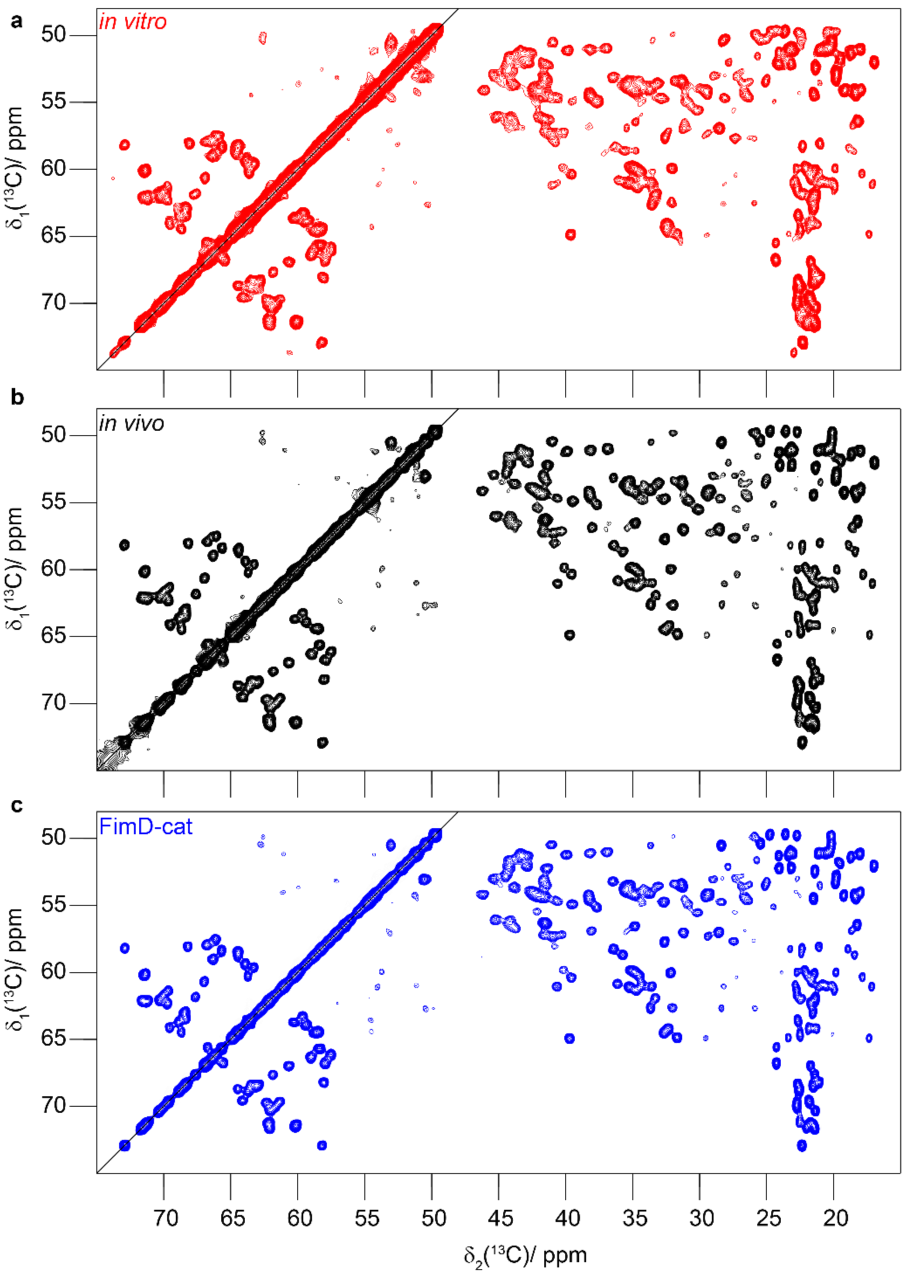


**Fig. S8: Solid-state NMR spectra of *in vitro*, *in vivo* and FimD-cat pili are highly resolved.**

**a-c** Aliphatic region of $^{13}C$-$^{13}C$ 20 ms DARR spectra of **(a)** *in vitro*-assembled pili, **(b)** *in vivo*-assembled pili, **(c)** and FimD-cat pili.

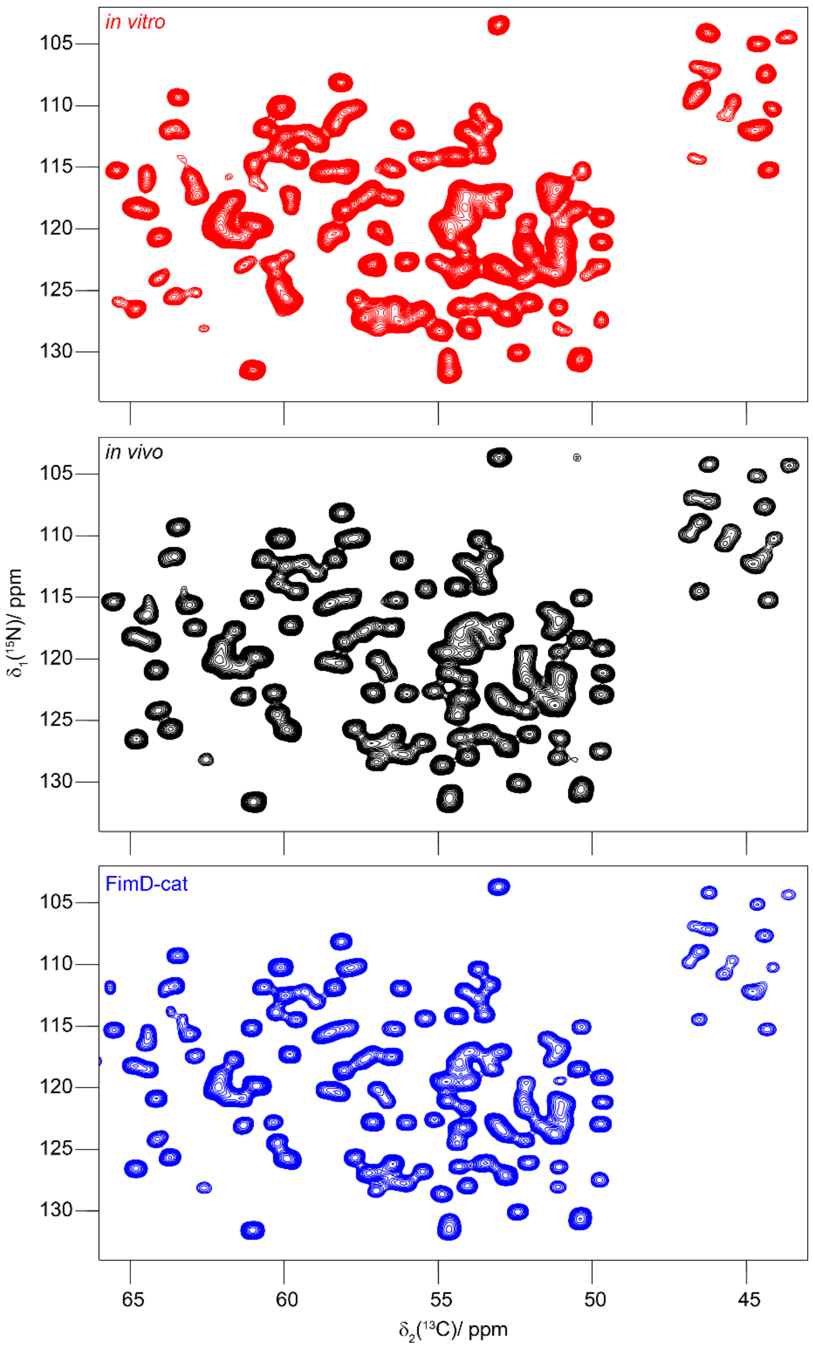


**Fig. S9: NCA spectra of FimD-cat pili and *in vivo*-assembled pili are identical.**

$^{15}N$,$^{13}C$ NCA spectra of *in vitro*-assembled pili (red), *in vivo*-assembled pili (black) and FimD-cat pili (blue).

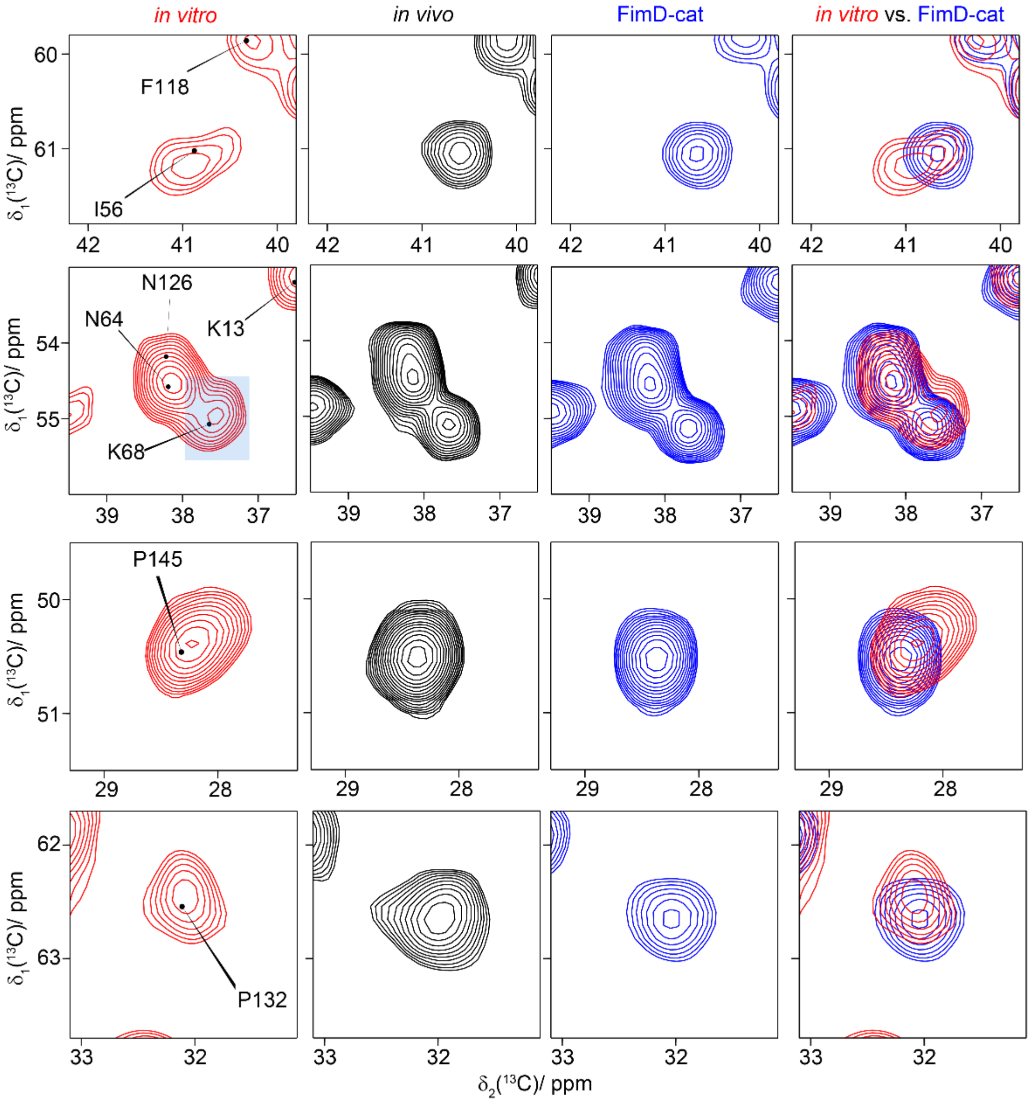


**Fig. S10: Minor chemical-shift changes observed between *in vitro* and *in vivo*-assembled pilus rods.**

Spectral fingerprints of 2D DARR spectra showing small chemical-shift changes for some residues in the *in vitro*-assembled (red) compared to the *in vivo*-assembled pili (black). FimD-cat (blue) pili yield identical spectra compared to the *in vivo*-assembled pili. Resonance assignments were taken from the BMRB database, accession code 25334 [2].

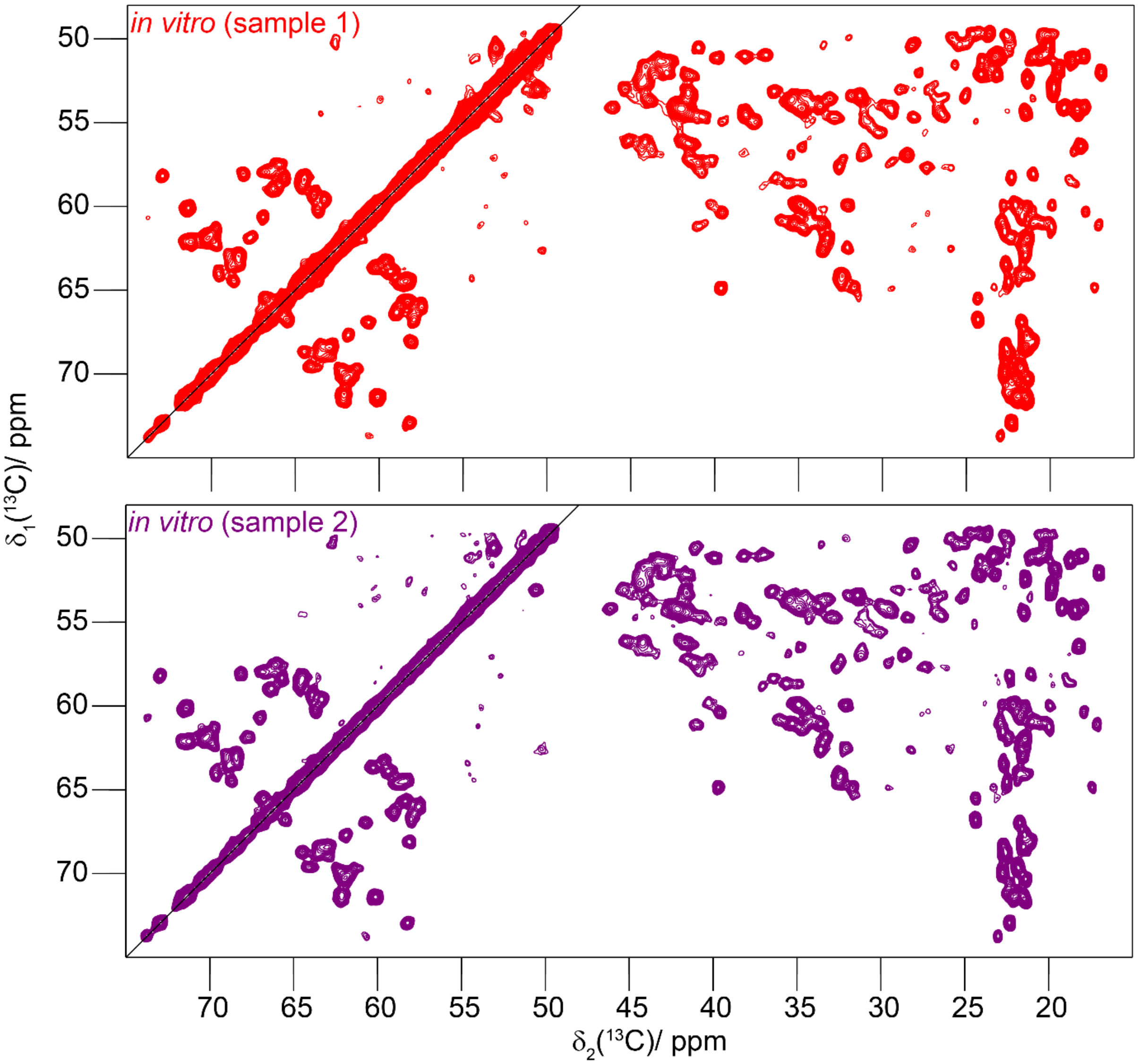


**Fig. S11: Two independent preparations of *in vitro*-assembled pili show similar solid-state NMR spectra.**

Aliphatic region of 2D DARR spectra of two independently produced *in vitro*-assembled pilus samples.

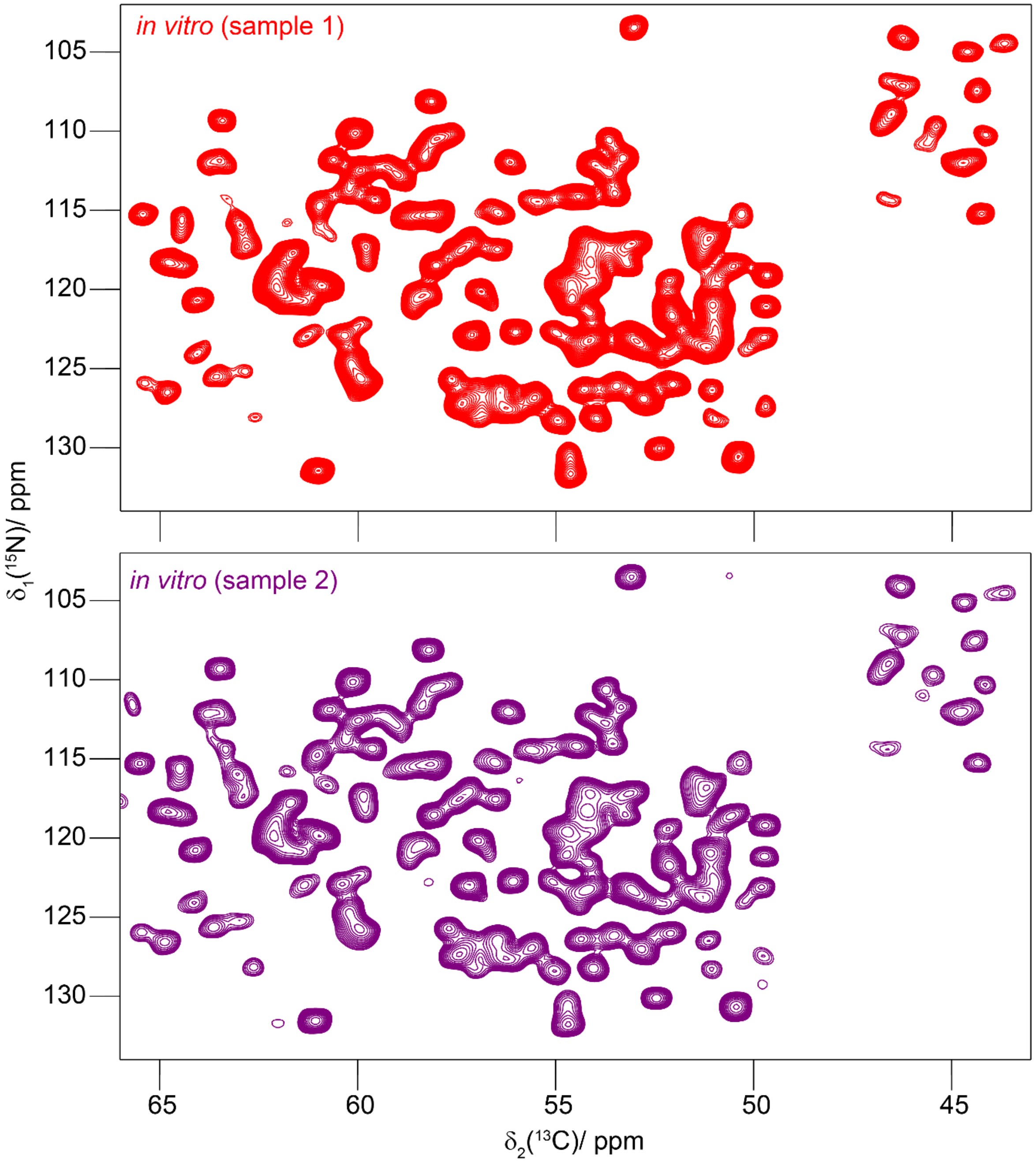


**Fig. S12: Two independent preparations of *in vitro*-assembled pili show similar solid-state NMR spectra.**

Aliphatic region $^{15}N$,$^{13}C$ NCA spectra of two independently produced *in vitro*-assembled pilus samples.

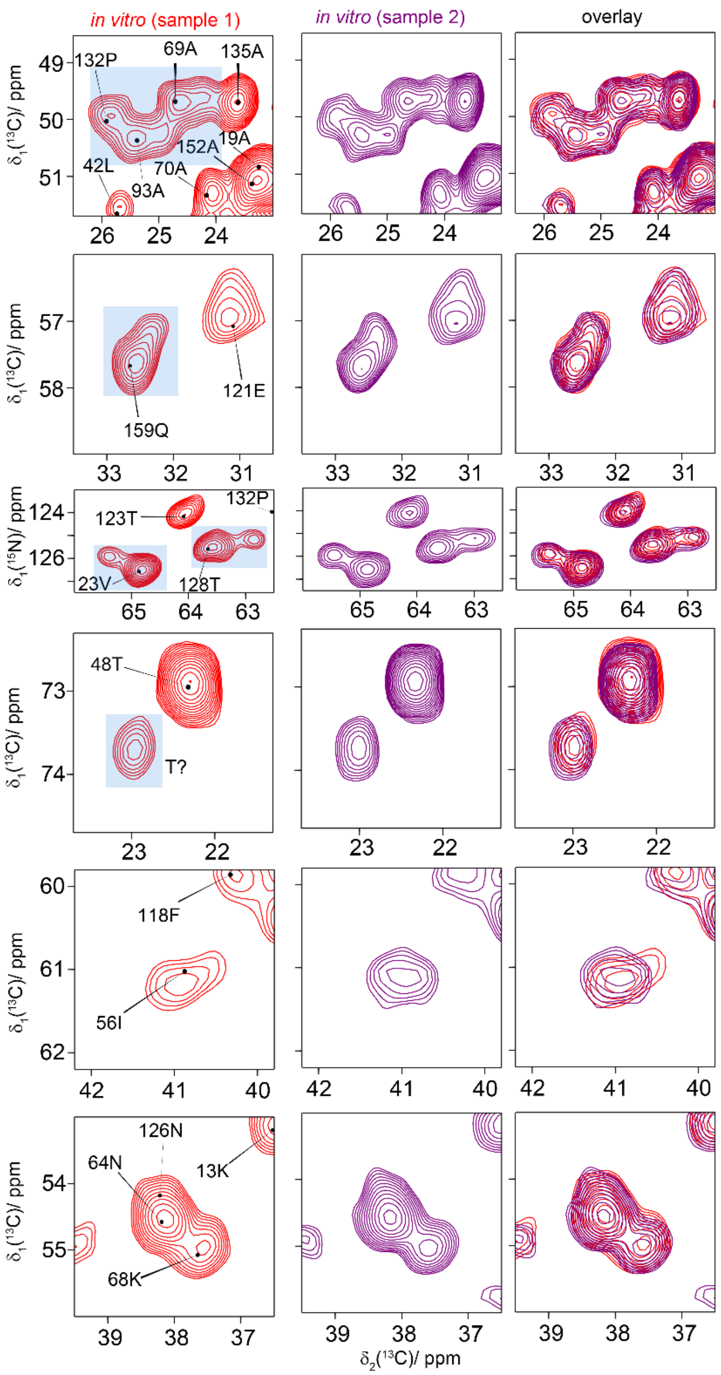


**Fig. S13: Two independent preparations of *in vitro*-assembled pili show similar solid-state NMR spectra.**

Spectral fingerprints taken from 2D DARR spectra of two independently produced *in vitro*-assembled pilus samples.

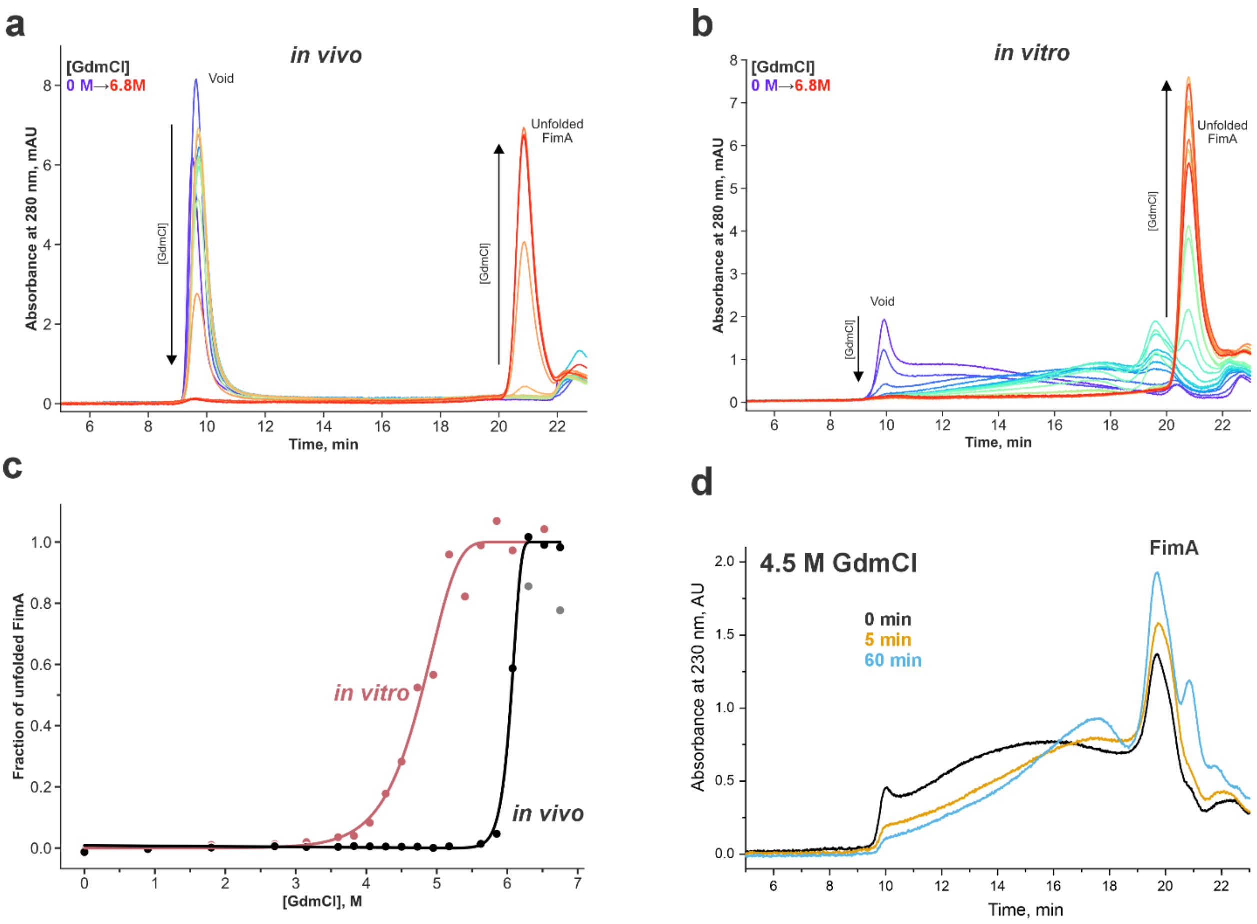


**Fig. S14: SEC experiments showing fragmentation and reduced stability of *in vitro*-assembled pili.**

**a, b** Analytical SEC profiles of **(a)** *in vivo* and **(b)** *in vitro*-assembled pili after incubation at increasing concentrations of GdmCl (pH 2.0, 25°C). Intact pili with a molecular mass above 5 MDa and longer than ~250 nm eluted at ~9.5 min (void volume). For *in vitro*-assembled pilus rods, fragmentation was observed already at low GdmCl concentrations, resulting in a broad tailing peak after the void volume peak. The peak corresponding to unfolded FimA (elution at ~21 min) is indicated. **c** Fraction of unfolded FimA from **(a)** and **(b)**, plotted against GdmCl concentration. *In vitro*-assembled (red) pili show much lower stability against GdmCl-induced unfolding than *in vivo*-assembled pili (black). The two grey data points are outliers in the dataset of *in vitro*-assembled pilus rods **d** Kinetics of *in vitro* pilus fragmentation at 4.5 M GdmCl. The chromatograms correspond to samples injected after incubation at 4.5 M GdmCl for 0 min (black), 5 min (yellow) and 60 min (blue).

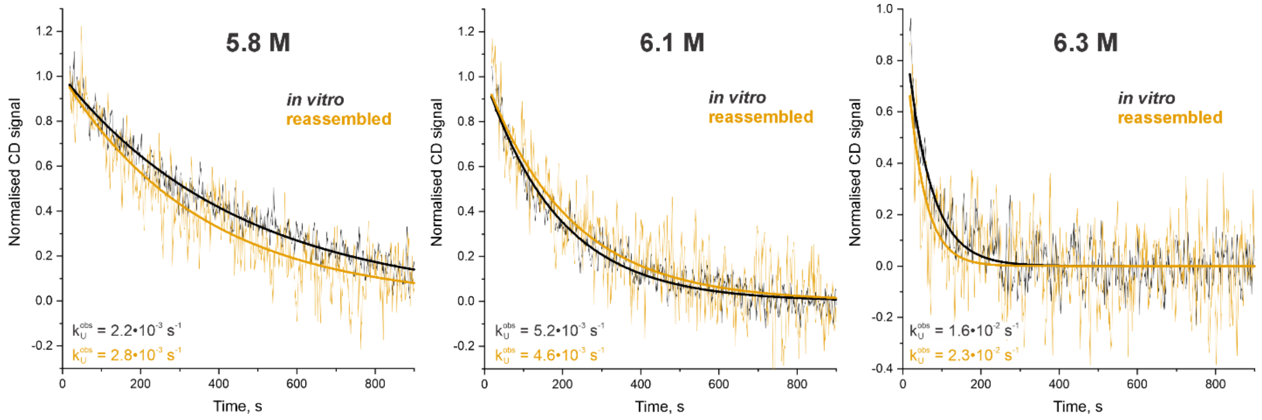


**Fig. S15: The stability against GdmCl-induced dissociation/unfolding of *in vitro*-assembled and reassembled pilus rods is very similar.**

*In vitro*-assembled pilus rods (black) and reassembled pilus rods after fragmentation (yellow) were mixed at pH 2.1 and 25°C in 5.8 M, 6.1 M, and 6.3 M GdmCl, dissociation/unfolding was monitored by the decrease in the CD signal at 230 nm. The derived rate constants ($k_U^{obs}$) for *in vitro* assembled pili and pili reassembled from fragments did not differ by more than a factor of 1.4.

## Supplementary References


1. Hospenthal, MK, Zyla, D, Costa, TRD, Redzej, A, Giese, C, Lillington, J, Glockshuber, R, and Waksman, G (2017) The Cryoelectron Microscopy Structure of the Type 1 Chaperone-Usher Pilus Rod. *Structure*, **25** (12), 1829-1838.e4, PMID: 29129382.

2. Habenstein, B, Loquet, A, Hwang, S, Giller, K, Vasa, SK, Becker, S, Habeck, M, and Lange, A (2015) Hybrid Structure of the Type 1 Pilus of Uropathogenic *Escherichia coli*. *Angew. Chemie - Int. Ed.*, **54** (40), 11691–11695, PMID: 26267365.

## Supplementary Tables

## Table S1 Kinetic stability parameters for all analysed pilus samples.

| ***In vitro*** | | | **FimD-cat** | | | ***In vivo*** | | |
|---|---|---|---|---|---|---|---|---|
| Parameter | Value | Error | Parameter* | Value | Error | Parameter* | Value | Error |
| $\ln(k_U^{H2O})$ | -34.34 | 0.81 | $\ln(k_U^{H2O})$ | -62.43 | 5.41 | $\ln(k_U^{H2O})$ | -99.15 | 10.85 |
| $m_U$ ($M^{-1}$) | 4.76 | 0.133 | $m_U$ ($M^{-1}$) | 8.46 | 8.19E-1 | $m_U$ ($M^{-1}$) | 13.78 | 1.62 |
| **$k_U^0$ ($s^{-1}$)** | 1.22E-15 | 9.88E-16 | **$k_U^0$ ($s^{-1}$)** | 7.67E-28 | 4.15E-27 | **$k_U^0$ ($s^{-1}$)** | 8.71E-44 | 9.45E-43 |
| **$k_U^{6.45M}$ ($s^{-1}$)** | 2.67E-2 | 1.53E-3 | **$k_U^{6.45M}$ ($s^{-1}$)** | 4.05E-4 | 2.55E-4 | **$k_U^{6.45M}$ ($s^{-1}$)** | 3.51E-5 | 2.69E-5 |
| **$t_{1/2}^{6.45M}$ (s)** | 26 | 4 | **$t_{1/2}^{6.45M}$ (s)** | 1709 | 1076 | **$t_{1/2}^{6.45M}$ (s)** | 19732 | 15132 |

* Obtained from linear regression of the $\ln(k_u)$ vs. [GdmCl] plots (Fig. 2c) between 5.9 and 6.8 M GdmCl

| **P pili *in vitro*** | | | **P pili *in vivo*** | | |
|---|---|---|---|---|---|
| Parameter | Value | Error | Parameter | Value | Error |
| $\ln(k_U^{H2O})$ | -40.02 | 2.01 | $\ln(k_U^{H2O})$ | -46.23 | 1.64 |
| $m_U$ ($M^{-1}$) | 4.84 | 0.29 | $m_U$ ($M^{-1}$) | 5.39 | 0.21 |
| **$k_U^0$ ($s^{-1}$)** | 4.16E-18 | 8.39E-18 | **$k_U^0$ ($s^{-1}$)** | 8.40E-21 | 1.38E-20 |
| **$k_U^{7.2M}$ ($s^{-1}$)** | 5.57E-3 | 2.38E-3 | **$k_U^{7.2M}$ ($s^{-1}$)** | 6.37E-4 | 1.88E-4 |
| **$t_{1/2}^{7.2M}$ (s)** | 125 | 53 | **$t_{1/2}^{7.2M}$ (s)** | 1088 | 320 |

## Table S2 Cryo-EM map and model statistics.

| Model | In vivo | In vitro | FimD-cat |
|---|---|---|---|
| Map statistics | | | |
| PDB/EMDB identifier | 6Y7S / EMD-10721 | 8PSV / EMD-17863 | 8PTU / EMD-17878 |
| Particles | 40k | 110k | 135k |
| B-factor | -49.97 | -45.07 | -46.49 |
| Resolution (Å) | 2.85 | 2.69 | 2.52 |
| Helical twist (°) | 115.0 | 115.0 | 115.0 |
| Helical rise (Å) | 7.85 | 7.76 | 7.80 |
| Box size (px) | 256 | 250 | 250 |
| Pixel size (Å) | 1.08 | 0.53* | 0.53* |
| Detector | Gatan K2 Summit | | |
| Dose | 50.4 | 97.54 | 49 |
| Number or recorded movies | 3468 | 3324 | 725 |
| Frames | 40 | 56 | 35 |
| Exposure (s) | 8 | 14 | 7 |
| Dose per frame (e $Å^{-1}$) | 1.25 | 1.74 | 1.4 |
| Model statistics | | | |
| Chains | 6 | 6 | 6 |
| Atoms | 6678 (Hydrogens: 0) | 6642 (Hydrogens: 0) | 6648 (Hydrogens: 0) |
| Residues | Protein: 954<br>Nucleotide: 0 | Protein: 948<br>Nucleotide: 0 | Protein: 948<br>Nucleotide: 0 |
| Bonds (RMSD) | | | |
| Length (Å) (# > 4sigma) | 0.002 (0) | 0.003 (0) | 0.003 (0) |
| Angles (°) (# > 4sigma) | 0.364 (0) | 0.426 (0) | 0.451 (0) |
| MolProbity score | 0.98 | 1.16 | 1.19 |
| Clash score | 2.06 | 3.68 | 4.06 |
| Ramachandran plot (%)[1] | | | |
| Outliers | 0.00 | 0.00 | 0.00 |
| Allowed | 0.85 | 2.03 | 1.60 |
| Favored | 99.15 | 97.97 | 98.40 |
| Rotamer outliers (%) | 0.00 | 0.00 | 0.00 |
| Data | | | |
| d FSC (half maps; 0.143) | 2.85 | 2.69 | 2.52 |
| d FSC model (0/0.143/0.5) | 2.5/2.8/3.0 | 2.4/2.6/2.7 | 2.4/2.5/2.6 |

* These data were acquired in super-resolution mode.

**Table S3**: Overview of resonances experiencing peak shifting, doubling and/or broadening when comparing various spectra of the *in vitro*- with the *in vivo*-assembled pili

| **Spectrum** | **Peak doubling and/or line broadening** | **Peak shifting** |
|---|---|---|
| $^{3}$C-$^{13}$C DARR | V17, A69, A72, A93, Q159 | A22, Q30, I56, K68, H82, P132, T144, P145, A150 |
| $^{15}$N-$^{13}$C NCA | V23, V37, I103, T128, F154 | L34, V52, Q57 |

**Table S4**: Intensity ratios of residues experiencing clear peak doubling in the two *in vitro* pili preparations.

| | Intensity ratio (sample 1) | Intensity ratio (sample 2) |
|---|---|---|
| V17 (CA,CB) | 1:1.6 | 1:1.2 |
| V23 (N, CA) | 1:2 | 1:1.7 |
| I103 (N,CA) | 1:1.7 | 1:1.8 |
| T128 (N,CA) | 1:1.5 | 1:1.7 |
